# Approaches to Enhance Natural Killer Cell-Based Immunotherapy for Pediatric Solid Tumors

**DOI:** 10.3390/cancers13112796

**Published:** 2021-06-04

**Authors:** Aicha E. Quamine, Mallery R. Olsen, Monica M. Cho, Christian M. Capitini

**Affiliations:** 1 Department of Pediatrics, University of Wisconsin School of Medicine and Public Health, Madison, WI 53705, USA; quamine@wisc.edu (A.E.Q.); mrolsen2@wisc.edu (M.R.O.); mcho54@wisc.edu (M.M.C.); 2 Carbone Cancer Center, University of Wisconsin School of Medicine and Public Health, Madison, WI 53705, USA

**Keywords:** NK cells, childhood cancer, solid tumor, neuroblastoma, Ewing sarcoma, rhabdomyosarcoma, osteosarcoma, hepatoblastoma, chimeric antigen receptor, CAR NK cells

## Abstract

**Simple Summary:**

Children with metastatic solid tumors typically have a very poor prognosis, especially when the cancer returns or is resistant to upfront treatment. There is hope that the cells in the immune system can be programmed to help patients recognize and eliminate their cancer. Natural killer (NK) cells are an important component of the immune system designed to eliminate virally infected cells and tumors. In the last 20 years, advances in cell manufacturing have allowed investigators to grow NK cells in the laboratory to high numbers and engineer the cells to become highly potent. While some clinical trials using NK cells in children with solid tumors have shown promise, we still have much to learn on how to use NK cells effectively. This review will discuss our current understanding of NK cell biology and its relevance to solid tumors that commonly affect children.

**Abstract:**

Treatment of metastatic pediatric solid tumors remain a significant challenge, particularly in relapsed and refractory settings. Standard treatment has included surgical resection, radiation, chemotherapy, and, in the case of neuroblastoma, immunotherapy. Despite such intensive therapy, cancer recurrence is common, and most tumors become refractory to prior therapy, leaving patients with few conventional treatment options. Natural killer (NK) cells are non-major histocompatibility complex (MHC)-restricted lymphocytes that boast several complex killing mechanisms but at an added advantage of not causing graft-versus-host disease, making use of allogeneic NK cells a potential therapeutic option. On top of their killing capacity, NK cells also produce several cytokines and growth factors that act as key regulators of the adaptive immune system, positioning themselves as ideal effector cells for stimulating heavily pretreated immune systems. Despite this promise, clinical efficacy of adoptive NK cell therapy to date has been inconsistent, prompting a detailed understanding of the biological pathways within NK cells that can be leveraged to develop “next generation” NK cell therapies. Here, we review advances in current approaches to optimizing the NK cell antitumor response including combination with other immunotherapies, cytokines, checkpoint inhibition, and engineering NK cells with chimeric antigen receptors (CARs) for the treatment of pediatric solid tumors.

## 1. Introduction

The innate immune system plays a critical role in establishing the first line of defense to nascent tumors on the basis of their expression of stress-induced ligands or cytokine stimulation. The discovery of a “natural” non-T killer cell responsible for low incidence of spontaneous malignant tumors in homozygous nude mice gave evidence to the importance of the innate immune effector cell known as natural killer (NK) cells [1], in antitumor cytotoxicity. Historically, NK cells were known as “third population cells” because they both lack the conventional surface antigen receptors of their T- and B- cell counterparts and mediate major histocompatibility complex (MHC)-independent killing. NK cells are heterogenous by nature; in the context of antitumor immunity, they serve a dual purpose of killing malignant cells and producing cytokines and chemokines to recruit the adaptive immune response [2]. With these characteristics, NK cells play a central role in tumor immune surveillance [3]. Yet, solid tumors are still able to develop despite an intact NK repertoire due to a variety of immune evasion strategies. Over the past 2 decades, several studies have uncovered ways to harness the potential of NK cells to target solid tumors and overcome common immune evasion strategies. Adoptive immunotherapy with ex vivo or in vivo activated NK cells has to date been used more commonly in adults with blood cancers, most recently with chimeric antigen receptor (CAR) NK cells for non-Hodgkin lymphoma. However, pediatric solid tumors remain a challenging target. 

Treatment for pediatric solid tumors is multimodal and combines cytotoxic chemotherapy, radiation, and surgery. The side effects of these treatments can persist into adulthood with long-term health effects. In an area where there is little room for further toxicity, the addition of effective cell therapy is a promising step to maximize targeted cytotoxicity with limited adverse effects. Unlike T cells, NK cells do not mediate graft-versus-host-disease (GVHD) and thus are an appealing option as an allogeneic, “off-the-shelf” therapy that could mediate anti-tumor responses without concern for long-term toxicity [4]. By gaining a better understanding of how the cumulative signaling of activating and inhibitory receptors expressed by NK cells contributes to dynamic regulation of their cytotoxicity response, we may leverage these mechanisms to maximize NK cell efficacy and limit any unintended short-term toxicity. 

## 2. NK Cell Activating and Inhibitory Receptors

**Figure 1:**
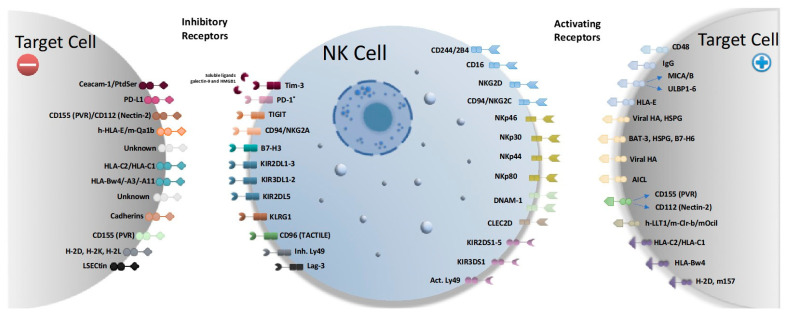
Activating and inhibitory NK cell receptors present in mice and humans. Abbreviations: PVR—polio virus receptor, HL—human leukocyte antigen, KIR—killer-cell immunoglobulin-like receptor, NKG2A—natural killer group 2A, NKG2D—natural killer group 2D, NKG2C—natural killer group 2C, DNAM-1—DNAX accessory molecule-1, LLT1—lectin-like transcript-1, HA—hemagglutinin, MICA/B—MHC class I chain-related protein A and B, ULBP-1—UL16 binding protein 1, AICL—activation-induced C-type lectin, PtdSer—phosphatidyl serine, Tim-3—T-cell immunoglobulin and mucin domain 3. Ceacam-1—carcinoembryonic antigen-related cell adhesion molecule 1, HMGB1—high-mobility group box 1, LSECtin—lymph node sinusoidal endothelial cell C-type lectin.

NK cells rely on a diverse set of activating and inhibitory receptors to appropriately direct their potent effector (Figure 1) [5]. In response to malignant cells, activating receptors on NK cells can induce apoptosis of the tumor or cause the NK cell to secrete cytokines. Therapies that stimulate these receptors may be exploited to treat pediatric solid tumors.

### 2.1. NK KIR Receptors

All nucleated cells express MHC class I (MHC-I) molecules on the cell surface, which is a critical feature that enables NK cells to execute immune surveillance. Tumor cells often downregulate expression of MHC-I to evade T cell recognition, however, low, altered, or missing MHC-I expression exposes tumor cells to NK cell recognition as NK cells are able to distinguish between self and non-self by binding with MHC-I through highly polymorphic receptors known as Ly49s in mice, and killer cell immunoglobulin-like receptors (KIRs) in humans. Murine Ly49 receptors are homodimeric type II transmembrane proteins containing C-type lectin-like domains (CTLDs) that are aptly named for their similarity to C-type lectins. However, unlike their carbohydrate binding cousins involved in Ca^2+^-dependent carbohydrate binding [6], Ly49 receptors cannot bind sugars [7]. This divergence allows Ly49 receptors to bind to unglycosylated MHC-I ligands [8,9]. Human NK cells do not express functional Ly49, but instead express the KIR homolog, which binds to HLA. KIRs are type I transmembrane glycoproteins. Similar to their Ly49 relatives, KIRs are highly polymorphic and polygenic, which contributes to the highly dynamic KIR genotype among individuals. Generally, activating KIR receptors have short cytoplasmic tails while inhibitory receptors have long cytoplasmic tails, which is reflected in KIR nomenclature. The first 2 digits in the KIR acronym describe the number of extracellular domains (2D or 3D) and the 3rd digit describes the length of the cytoplasmic domain or whether the KIR is a pseudogene (S,L, or P). KIRs are further differentiated by number and asterisk, which signify allelic variants [10]. 

Despite their structural dissimilarity, Ly49 has provided a useful model system to study the role of MHC-I recognition in immune regulation because KIRs are functionally similar to Ly49s in terms of MHC-I recognition. Activating receptors such as Ly49s and most KIRs (except KIR2L4) transduce signals by the immunoreceptor tyrosine-based activation motif (ITAM)-containing adapter protein, DAP12, through a charged residue in their transmembrane domain. Upon activation, the DAP12 ITAM recruits a Src family kinase to phosphorylate the ITAM, which mobilizes and activates the Syk family kinases (Syk/Zap-70) [11]. Inhibitory Ly49s and KIRs lack the ITAM and instead transduce their signal through a single immunoreceptor tyrosine-based inhibition motif (ITIM) domain in the cytoplasmic tail [12]. When engaged, the ITIM becomes tyrosine-phosphorylated, which leads to the recruitment and activation of inhibitory signaling molecules including tyrosine phosphatases Src homology 2 (SH2) domain-containing phosphatases-1 and -2 (SHP-1 and SHP-2) and SH2 domain-containing inositol polyphosphate 5-phosphatase (SHIP) [13]. Inhibition of NK cell activation favors the recruitment of SHP-1 to the ITIM, which is expressed in inhibitory receptors such as NKG2, Ly49 and KIR receptors [14,15,16,17]. In addition to independent signaling, ITIMs can exert an inhibitory signal by colocalizing with activating receptors. Cross-linking of these inhibitory receptors to an activating receptor complex can augment inhibitory signaling [18]. Co-ligation of the ITIM to the activating receptor allows the ITIM to be in close proximity to the Src family kinase, which facilitates local phosphorylation of the ITIM. This downstream regulatory mechanism enables inhibitory signals supersede and suppress activation. It has been demonstrated that activating Ly49/KIR receptors have lower affinity to their ligands compared to inhibitory receptors to prevent autoimmunity [19]. In mice, activating Ly49D and Inhibitory Ly49s (Ly49A/C/G) share the H2-Dd MHC-I molecule as a ligand; in humans, inhibitory receptor KIR2DL2/KIR2DL3 and activating receptor KIR2DS2 share the same ligand, HLA-C1. This duality is vital for ensuring that NK cells will not become autoreactive, yet such complexity makes the MHC-I system difficult to leverage by way of targeting specific receptors with agonists or antagonists. MHC-I mediated activation of NK cell killing relies on a higher expression frequency and culmination of activating signals over inhibitory signals, yet expression of just two inhibitory KIR can significantly increase NK cell resistance [20].

Ly49/KIR receptor interactions also regulate NK cell functional competency through a “licensing” process, known as NK cell education, which results in self-tolerance [21,22]. Following licensing, NK cell reactivity is amplified in correlation with the number of self-MHC-I-specific inhibitory receptors, whereas NK cells that are lacking self-reactive receptors, such as KIRs, are anergic [10]. These findings have revealed that NK cell inhibitory receptor/ligand pairs that may represent potential targets for checkpoint blockade in cancer immunotherapy. However, blocking inhibitory KIR receptors in NK cells may not be advantageous. KIR^-^ NK cells adopt a mature yet hypo-responsive phenotype, unable to respond to ADCC or lyse tumor targets as efficiently as wild type (WT) NK cells [22,23]. This data aligns with the “calibration model” that suggests that recognition of inhibitory self-MHC-I stimuli are important for “licensing” NK cell reactivity, possibly due to crosstalk between activating and inhibitory pathways that lead to increased efficiency in the activating transduction circuits [23]. 

Koh and colleagues showed preclinical data indicating that Ly49C+I blockade can enhance NK cytotoxicity after syngeneic hematopoietic stem cell transplant (HSCT) against murine leukemia, T cell lymphoma, and mastocytoma in vitro and murine leukemia in vivo [24]. However, clinical efficacy of KIR blockade has yet to show consistent benefit. Lirilumab (IPH2101), a novel anti-inhibitory KIR antibody designed to prevent NK cell recognition of MHC-I inhibitory signals, was investigated in a phase 2 clinical trial (NCT01687387) for adult acute myeloid leukemia (AML) where it failed to show statistical improvement of leukemia free survival [25]. A phase 1 efficacy study of Lirilumab in patients with multiple myeloma found that infusion of Lirilumab resulted in an unexpected decrease of NK cell responsiveness and KIR2D expression on the NK cell surface though trogocytosis (NCT01248455) [26]. To date, no studies have been performed in children with solid tumors. 

**Figure 2:**
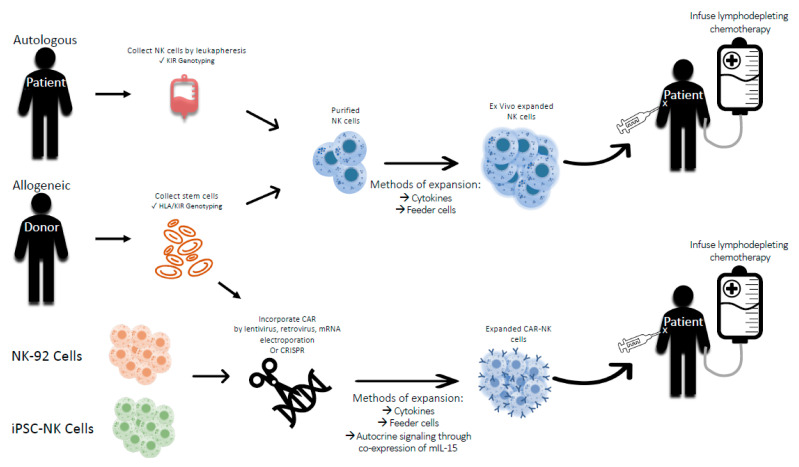
Approaches to clinical applications of adoptive NK cell therapy. Sources of NK cells may originate from an autologous donor or allogeneic donor, which will influence how they are collected. Peripheral blood mononuclear cells from autologous donors are collected using leukapheresis and may be subject to KIR genotyping, whereas peripheral blood mononuclear cells from allogeneic donors may be subject to HLA and KIR genotyping. Other allogeneic sources of NK cells include NK cells from cord blood, a cell line such as NK-92 cells, or NK cells derived from induced pluripotent stem cells (iPSC). These sources of NK cells offer the benefit of being an “off-the-shelf” adoptive cell therapy. NK cells require expansion and activation through cytokines, such as IL-2, IL-12, IL-15, IL-18, or IL-21. Membrane bound (mb) IL-15 or IL-21 with 41BB ligand have been genetically engineered into feeder cell lines, such as K562 cells, to generate robust NK expansions. NK cells can be genetically modified to express CARs that enable them to target a specific antigen. Recent developments in CAR NK technology have found that co-expression of IL-15 enhances CAR NK persistence, therefore obviating the need for systematic administration of IL-15. Abbreviations: HLA—human leukocyte antigen, KIR—killer immunoglobulin-like receptor, CAR—chimeric antigen receptor.

Instead, allogeneic hematopoietic stem cell transplant (HSCT) and adoptive cell therapy with donor-derived, allogeneic NK cells (Figure 2) are attractive alternatives to augment the graft-versus-tumor effect of NK cells generated by KIR mismatch [27,28]. While there are several studies observing the benefit of KIR-mismatched NK cells in leukemias [29,30,31,32,33,34,35], more clinical and preclinical studies are needed to demonstrate the benefit of KIR mismatch in pediatric solid tumors. Delgado et al. [20] found that osteosarcoma cell lines were most sensitive to allogeneic NK cell lysis when KIR receptor-ligand incompatibility was maximized. The osteosarcoma cell line lacking expression of HLA-C group 1 and 2 was efficiently lysed by NK cells from 2 donors lacking expression of KIR2DL1 or 3 major inhibitory KIRs (KIR2DL1, KIR2DL2/3, and KIR3DL1), respectively. Osteosarcoma cell lines with high MHC-I expression were significantly less susceptible to NK cell cytolysis by NK cells from either donor. However, after passage-induced down regulation of HLA-C group 1, 2 and HLA-Bw4 susceptibility to apoptosis by donor NK cells significantly increased. Activating KIR gene content has been associated with increased NK potency against a panel of pediatric tumors including Ewing sarcoma, rhabdomyosarcoma, neuroblastoma, lymphoma, leukemia, and brain tumors suggesting that KIR mismatch alone is not enough to predict efficacy of NK cell adoptive therapy [36].

Low MHC-I expressing pediatric tumors are an ideal target for NK cell adoptive therapies because target cells that have reduced self-MHC-I expression cannot deliver the necessary negative signal and are susceptible to NK cell lysis, as outlined in the “missing-self” hypothesis [37]. MHC-I downregulation is a common mechanism of tumor immune escape by which tumors evade T cell recognition. Low MHC-I expression has been reported in neuroblastoma and Ewing’s sarcoma where it is associated with high-risk patients and disease progression, respectively [38]. Studies have also shown that poorly differentiated rhabdomyosarcomas are negative for classical HLA Class I expression [39]. NK cell-based therapies could be a potential strategy for those tumors that demonstrate low or absent expression of HLA.

### 2.2. NK Activating Receptors

#### 2.2.1. NKG2 Receptor Family

The NKG2 family, CD16/Fc gamma RIIIA, DNAM-1, and natural cytotoxicity receptors (NCRs) NKp30, NKp44, and NKp46 are crucial receptors for NK cell mediated targeting of low MHC-I tumors, and these receptors have been targeted in preclinical studies of pediatric solid tumors. NKp30 and NKp46 have been implicated in NK cell-mediated cell lysis of both neuroblastoma cell lines and freshly isolated neuroblasts [40]. Compared to the other NCRs, NKp46 expression is uniquely specific to NK cells, and it is the only NCR that is evolutionarily conserved in humans and mice suggesting its importance as the primary NCR [40]. NKp46 clusters at the immunological synapse, which causes the required accumulation of F-actin for a functional synapse [41]. Multiple groups [42,43,44] have established the ability of NK cells to eliminate tumor metastasis through NKp46. Glasner and colleagues demonstrated that the mechanism behind NKp46 tumor elimination occurs though a combined pathway involving the secretion of IFN-γ and editing of tumor architecture [43].

The NKG2 receptor family are C-type lectin type II transmembrane proteins composed of both activating and inhibitory isoforms. Inhibitory NKG2A forms heterodimers with CD94 and recognizes nonclassical MHC-I molecule, human leukocyte antigen (HLA)-E [45]. Ligand binding prompts downstream signals that are transmitted by an ITIM, aligning NKG2A with other inhibitory NK cell receptors. Activating NKG2C and NKG2E lack the ITIM but are able to heterodimerize with CD94 and recognize HLA-E, which exemplifies one of several instances where NK cell receptors with opposing functions share ligands. NKG2C and NKG2E signal through the DAP12/KARAP adaptor molecule; however, studies suggest NKG2E does not reach the cell surface and instead functions as an intracellular protein [46,47].

Adaptive or memory NK cells are known to be rapidly expanded in response to cytomegalovirus (CMV) infection and characterized by high NKG2C expression [48,49]. CMV reactivation has been associated with decreased risk of disease progression (DP) post-transplant leukemia patients, which prompted investigation into whether adoptive therapy with adaptive NK cells established any clinical benefit. The antitumor potency of NKG2C+ NK cells was confirmed in an in vitro study in which adaptive NK cells from cytomegalovirus-infected children after transplant were cocultured in vitro with K562 tumor targets [50]. NKG2C+ NK cells were detectable past 3 months and effectively killed HLA-E expressing tumor targets despite their poor response to IL-12/IL-18 and low production of interferon-γ (IFN-γ). Clinical studies showed that therapy with adaptive NKG2C+ NK cells was associated with lower DP after transplant in a study of adult patients with advanced acute leukemia [51,52]. However, the clinical benefit of NKG2C+ NK cells in leukemia has been disputed by other groups [53] and no studies to date have targeted this receptor in pediatric solid tumors indicating the need for further investigation.

NKG2D plays an important role in enhancing NK cell activity against Ewing sarcoma, neuroblastoma, osteosarcoma, and rhabdomyosarcoma [54,55,56,57,58]. When NK cells are activated, NKG2D forms a complex with a signaling adaptor, DNAX-activating protein 10 (DAP10), which in turn activates the PI3K–Akt pathway for cytotoxicity [59,60]. NKG2D in humans recognizes stress ligands, MHC-I chain-related molecule (MIC) A/B, ULBP families, and several retinoic acid early transcripts 1 (RAE-1) ligands. Mouse NK cells express two NKG2D isoforms; NKG2D-Long (L) that binds with DAP10, and NKG2D-Short (S) that binds with DAP10 and DAP12. Mouse NKG2D binds H-60, Rae1 ligands, and mouse ULBP-like transcript 1 (Mult-1) [61]. After “priming” with IL-2/IL-15, NK cells stimulated with MICA and ULBP generated smaller and larger clusters of NKG2D respectively in a ligand specific manner [62]. MICA can strongly downregulate NKG2D more than ULBP [63]. Tumor cells are more likely to express NKG2D ligands under conditions of stress, making NKG2D an attractive potential therapeutic target. Targeting tumors based on their expression of NKG2D ligands with primed NK cells may result in differing responses depending on the tumor expression profile.

#### 2.2.2. DNAM-1

The adhesion molecule, DNAM-1, is another receptor known to be critical for NK cell activation due to its synergizing effect on activating receptors increasing actin polymerization and granule polarization [64]. In NK cells, DNAM-1 triggers direct cytotoxicity upon interaction with its ligands, PVR/CD155 and Nectin2/CD112 [65]. However, new insights on the DNAM-1+ NK cell populations provide a deeper glimpse into how DNAM-1 expression controls NK cell function relevant to targeting this subpopulation. DNAM-1^+^ NK cells contained more IFN-γ-producing cells, produced higher levels of inflammatory cytokines, and were more effective at controlling rhabdomyosarcoma tumor growth and experimental metastasis than DNAM-1^−^ NK cells [66]. Neither DNAM-1 blockade nor anti-CD155 inhibition impacted cytokine production by DNAM-1^+^ and DNAM-1^−^ NK cells, suggesting that increased cytokine production is a unique characteristic of DNAM-1^+^ NK cells but not dependent on DNAM-1^+^ interaction with CD155. While this suggests DNAM-1^+^ NK cells may have broader application due to the potent effector function shown regardless of whether or not the tumor target expresses CD155 or CD112, neuroblastoma cells that express CD155 display the highest susceptibility to NK-mediated killing [40]. Thus, CD155 may be a potent target to enhance NK cell cytotoxicity against neuroblastoma through the DNAM-1 pathway. With respect to other solid cancers, Ewing sarcoma and rhabdomyosarcoma are also susceptible to NK-mediated cytotoxicity that is dependent on NKG2D and DNAM-1 signaling [67,68,69].

#### 2.2.3. CD16

FcγRIIIa (CD16) is a critical receptor for NK cell activation; however, it is unique because target cell lysis is achieved through antibody dependent cellular cytotoxicity (ADCC), rather than direct cytolysis or cytokine production. This mechanism, which allows NK cells to target specific tumor antigens, underscores NK cells as the ideal candidate for adoptive therapy combined with monoclonal antibodies. CD16 has low-affinity for the Fc region of IgG antibodies, and it is sufficient alone to trigger activated, resting, and non-educated NK cells to carry out ADCC responses [70]. Multiple preclinical studies have shown the benefit of combining NK cells with cytotoxic monoclonal antibodies to enhance the ADCC response. Yet, the efficacy of ADCC relies on the expression profile of NK cells, which is influenced by both genetic and environmental factors. For example, the FcgRIIIa-158F/V polymorphism was first identified 1997 and shown to produce a CD16 phenotype associated with enhanced receptor binding in response to a standard stimulus [71,72]. In this context, FcγRIIIa polymorphisms may affect the response of therapeutic monoclonal antibodies that feature the human constant Fc IgG1 region. Weng WK et al. [73] reported the FcγRIIIa V/V allotype to be correlated with better response to rituximab in patients with untreated/relapsed follicular lymphoma [74]. More studies are paramount to understand how to mobilize CD16^+^ NK cell populations and optimize therapeutic monoclonal antibodies augment affinity for FcγRIIIa. Nevertheless, ADCC is an important mechanism for targeting pediatric solid tumors by targeting overexpressed tumor antigens on the cell surface, such as the disialoganglioside GD2. GD2 is ubiquitously on neuroblastoma and osteosarcoma cells, and is FDA approved for the upfront treatment of neuroblastoma as a maintenance therapy. Yet clinical trials with osteosarcoma have not been successful [75], which could reflect different tumor microenvironments or our lack of understanding of the role of CD16^+^ NK cell subsets in controlling these tumors by ADCC. 

### 2.3. NK Inhibitory Receptors

#### 2.3.1. PD-1/PD-L1

Programmed death-ligand 1 (PD-L1) is a transmembrane protein that is commonly expressed on several pediatric solid tumors. A retrospective study of 500 pediatric cancer patients found PD-L1 expression at varying degrees in neuroblastoma (18.9%), Ewing’s sarcoma (7.7%), osteosarcoma (6.7%), and rhabdomyosarcoma (5.6%), and in neuroblastoma high PD-L1 expression was significantly correlated with a higher risk of relapse [76]. Tumor expression of PD-L1 is partially induced by IFNγ [77]. This mechanism may be controlled in PD-L1 expressing pediatric tumors by combining NK cell treatment with PD-1 checkpoint inhibitors, although the expression of PD-1 on NK cells remains controversial [78]. Although there are preclinical studies that show the benefit of immune checkpoint inhibition, there are currently no FDA approved PD-L1 inhibitors for pediatric solid tumors [77,79,80,81]. Nivolumab and pembrolizumab have been used in phase 1 and 2 trials for pediatric patients with relapsed malignancies, but only pembrolizumab is approved for use in children with mismatch repair deficiency/microsatellite instability or Hodgkin lymphoma. Nivolumab has shown efficacy against neuroblastoma in preclinical models and in case reports when combined with anti-GD2 monoclonal antibodies but has not been proven effective as monotherapy in solid tumors [82,83,84]. Nivolumab is currently being investigated in combination with anti-GD2 monoclonal antibody and systemic radiation therapy with MIBG therapy (NCT02914405). Further exploration is needed to determine the role of PD-1 inhibition, alone or in combination with NK cells, in treating pediatric solid tumors. There is evidence in preclinical models of adult head and neck squamous cell carcinoma (HNSCC) and non-small-cell lung cancer (NSCLC) that anti-PD-L1 monoclonal antibodies enhance NK cell mediated ADCC [85]. However, this has not yet been investigated in pediatric solid tumors that may have lower PD1 expression. 

#### 2.3.2. CD94/NKG2A

The CD94/NKG2A heterodimer (NKG2A) is a C-type lectin receptor that recognizes HLA Class I E molecules in humans and Qa-1b in mice. NKG2A is expressed early during NK cell differentiation, preceding KIR expression. NKG2A binding to its ligand results in inhibitory signaling transduced through ITIMs that suppresses cytokine secretion and cytotoxicity. Kamiya et al. found that protein expression blocker-induced downregulation of NKG2A on NK cells increased cytotoxicity against HLA-E expressing tumor cell lines in vitro [86]. Solid tumors can upregulate HLA-E expression to escape NK killing, and HLA-E upregulation correlates with poor prognosis [87]. Tumor infiltrating NK cells also express higher frequencies of NKG2A [88]. Neuroblastoma with low classical MHC-I expression have higher non-classical MHC-I expression including HLA-E [89]. Monalizumab, a monoclonal antibody that blocks NKG2A, has been shown to improve NK cell function and tumor infiltration that is synergistic with anti-PD-L1 treatment [90,91]. To date, monalizumab has not been tested in pediatric solid tumors.

#### 2.3.3. TIGIT

TIGIT is an Ig superfamily receptor that is expressed on T cells and NK cells. Upon interaction with its ligands, including CD155 and CD112, it downregulates immune cells by attenuating NK cell degranulation, cytokine production, and cytotoxicity [92]. CD155-TIGIT ligation results in decreased microtubule organization center movement to the immunological synapse of the NK cell resulting in defective degranulation and inhibits Pi3K and MAP kinase activation [93]. TIGIT inhibits human NK cytotoxicity against the tumor by interacting with CD155 and CD112 and does so by an “alternative self” mechanism by preventing self-killing despite activating DNAM-1 signal [94]. Due to its higher affinity for CD155, TIGIT outcompetes DNAM-1, resulting in inhibitory signaling and preventing DNAM-1 signaling. Thus, TIGIT presents an attractive target to enhance NK cell antitumor cytotoxicity.

In mouse melanoma models, tumor-infiltrating NK cells have higher TIGIT expression than NK cells in surrounding tissues, and TIGIT^+^ NK cells had lower IFNγ, TNF, CD107a, and TRAIL expression and exhibited greater apoptosis, demonstrating an exhausted phenotype, which could be relieved in TIGIT^−/−^ tumor recipients or anti-TIGIT antibody blockade. Further, TIGIT blockade and rechallenge with melanoma resulted in potent antitumor memory response, possibly by the effect of NK cells enhancing CD8^+^ T cell immunity [95]. While in murine preclinical models anti-TIGIT monoclonal antibodies have limited efficacy to treat subcutaneous tumors, but the combination of TIGIT blockade and PD-1/PD-L1 pathway inhibitors is promising. While in murine preclinical models anti-TIGIT monoclonal antibodies have limited efficacy to treat subcutaneous tumors, but the combination of TIGIT blockade and PD-1/PD-L1 pathway inhibitors is promising. This approach has been extended to treating glioblastoma in conjunction with anti-PD-1 therapy in mouse preclinical models [96]. This combination therapy is being tested in a phase I clinical trial for adults with glioblastoma (NCT04656535) and potentially could be extended to pediatric patients with high grade glioma. Tiragolumab, an anti-TIGIT human IgG1, has breakthrough therapy designation by the FDA for treatment of metastatic non-small cell lung cancer (NSCLC) in combination with anti-PD-L1 therapy [97].

#### 2.3.4. TIM-3

NK cells also constitutively express T-cell immunoglobulin and mucin domain 3 (TIM-3). TIM-3 binds to Ceacam-1 and phosphatidyl serine on target cells and soluble ligands galectin-9 and HMGB1 [98]. On NK cells, TIM-3 expression increases following cytokine stimulation and promotes IFN-g production [99]. Although increased TIM-3 expression correlates with T cell dysfunction, on NK cells, NK cells with high TIM-3 expression are highly responsive and robustly degranulate against target cells. However, TIM-3 crosslinking on NK cells can inhibit NK cell cytotoxicity induced by activating NKG2D and CD16 receptors, suggesting that its function is context dependent [100]. Currently, in preclinical research and Phase I clinical trials, TIM-3 inhibitors are administered as T cell response enhancers to treat advanced solid tumors, and these therapies are likely to have various consequences for NK cell activity as well. TIM-3 inhibitors have also been combined with PD-L1 inhibitors in adult patients with relapsed solid tumors (NCT03099109), however trials in pediatric solid tumors have not yet been developed [101]. 

#### 2.3.5. LAG-3

Lymphocyte activation gene-3 (LAG-3) is an inhibitory receptor expressed on NK cells and T cells that bind to MHC Class II and lectin [102]. Its proposed ligand is LSECtin, and its effects on NK cell function are largely unknown [102]. Among limited evidence, NK cells from LAG-3 deficient mice show reduced killing against some tumor targets, though cytotoxicity against MHC-deficient blasts is preserved [103]. While in murine NKs, anti-LAG-3 antibodies reduced NK-mediated killing of Yac-1 cells, this has not borne out with human NK cells [104]. Further investigation into the role of this receptor is needed to determine its impact on NK cell function and antitumor potential, especially since anti-LAG3 antibodies like eftilagimod alpha and relatlimab are in clinical testing (Table 1).

#### 2.3.6. B7-H3

B7 homolog 3 protein (B7-H3), also known as CD276, is a B7 superfamily molecule that is expressed in the tumor microenvironment and has coinhibitory function on immune effector cells, most prominently decreasing T cell function. In T cells, it has costimulatory and coinhibitory regulatory functions, and co-stimulation occurs by B7-H3 binding to TLT-2 expressed on T cells. Aberrant B7-H3 expression is associated with poor outcome in various malignancies [87]. In mice, B7-H3 silenced glioma cells resulted in tumor formation but decreased metastasis [105]. It also directly inhibits NK cell activation and suppresses NK-cell mediated control of tumor growth and antitumor responses [106]. Specifically, in tissue cultures, B7-H3 inhibits NK cell activity, and studies have shown that soluble and cell surface B7-H3 inhibit NK cell cytotoxicity [107]. However, the receptor expressed on NK cells that binds to B7-H3 is unknown. With respect to targeting B7-H3, bispecific killer cell engager treatment and monoclonal antibodies are currently being explored (see Table 1), with one trial recently completed in pediatric solid tumors (NCT02982941).

## 3. Cytokines

To date, there are a large number of in vitro studies that use cytokine-mediated NK cell expansion to upregulate NK cell activating receptors to tip the balance of NK cell reactivity towards tumor cells. NK cell development is tightly regulated through cytokines that signal through the common gamma chain, including interleukin 2 (IL-2), IL-4, IL-7, IL-9, IL-15, and IL-21. IL-15 is the primary cytokine responsible for NK cell propagation, enhanced survival, and cytolytic function. Upon ligation, IL-15 signaling uses Janus-associated kinases (JAK) and signal transducer and activator of transcription (STAT) signaling to initiate cellular activation [116]. The impact of IL-15 on NK cells has been leveraged against a several pediatric tumor types. For example, rhabdomyosarcoma (RMS) cell lines were shown to be significantly more susceptible to IL-15 treated allogeneic NK cells than resting NK cells [58]. DNAM-1 and NKG2D expression was critical for lysis, however the expression level of their corresponding ligands did not correlate with the efficacy of NK cell-mediated tumor lysis, and blockade only reduced cytotoxicity by 25–50%. However, IL-15 activated NK cells had increased expression of NCRs, which may explain the sustained effector function. These results are consistent with studies utilizing Ewing sarcoma and osteosarcoma [67,117]. NK cell treated with IL-12, IL-18, and IL-15 had hindered degranulation, but produced a higher percentage of IFN-γ positive NK cells than NK cell treated with IL-15 alone (100% of cells, compared to less than 3% respectively). These cytokine-induced NK cells exhibit memory-like behavior, with enhanced responses upon re-stimulation with cytokines or through activating receptors against AML in preclinical studies [118]. IL-15/IL-18+IL-12 activated NK cell may have a supportive role in transplantation immunotherapy. In mouse models of mismatched hematopoietic transplant, they induce a protective effect against acute GVHD by inhibiting proliferation of donor T cells [119]. In the context of pediatric cancer, other signaling molecules included in ex vivo NK cell activation is 41BBL, IL-2, and IL-21 [120]. 

## 4. Canine Models of NK Adoptive Immunotherapy for Pediatric Solid Tumors

A downside to many preclinical immuno-oncology models is the lack of similarity in tumors developing spontaneously with an intact immune system. This hampers the translation from in vitro and mouse in vivo models to human patients. However, canine models can serve as a bridge between preclinical and human trials, particularly for dosing of children due to potential overlaps in weight. NK cell therapy in dogs has been investigated but has faced considerable challenges. NK cells are not as well defined in dogs as they are in humans. They are generally identified as CD3-, CD4-, NKp46+, CD5^dim^ non-B, and non-T cells [121]. Unlike human NK cells, canine NK cells do not express CD56 [122]. Further research with canine NK cells is complicated by a lack of antibodies and other research tools aimed at identifying canine homologs of NK markers. Multiple groups have made significant strides in canine NK cell definition and expansion. The Canter Lab has shown that NK cells can be grown and isolated from peripheral blood of healthy dogs and osteosarcoma patients [123]. Similar to human NK cells, canine NK cells can be grown with irradiated feeder cell lines, such as genetically modified K562 cells or with cytokines including IL-2/12/15/18/21. Canine NK cells demonstrated cytotoxicity in vitro and show increased expression of Granzyme B [124].

For osteosarcoma, companion dogs are very comparable to pediatric patients since it is a common disease of both canine and pediatric patients [125]. Dogs develop spontaneous osteosarcomas with an incidence of ten times that of humans, thus dogs are an ideal model for pediatric osteosarcoma, representing a tumor developing spontaneously [126]. In both species, metastatic and relapsed disease have poor survival with current chemotherapy. Immunotherapy is a promising area for osteosarcoma treatment [117,127]. Osteosarcoma is chemotherapy and radiation resistant, and it is primarily treated surgically [127]. Pediatric and AYA patients typically undergo neoadjuvant chemotherapy, followed by local surgical control, with more chemotherapy following [128]. Canine patients typically have surgery as the first treatment with or without chemotherapy after. Radiation is generally reserved for palliation of lung metastases. If possible, lung metastases are surgically resected as well. However, survival, particularly in metastatic disease, is poor in both species. Pediatric patients without metastases have a survival of 60–70%, but with metastatic disease the survival drops to 10–30% at 5 years [127]. Of dogs who present with locally aggressive disease, 85% have metastatic disease to the lungs within 6 months, given palliative radiation therapy alone [123].

Currently the Canter Lab is leading a first-in-dog trial using NK cells for patients with osteosarcoma at University of California Davis. Dogs are eligible with locally advanced but non-metastatic osteosarcoma, and owners are not pursuing amputation (standard of care) or chemotherapy. The study combines two weekly autologous NK cell intralesional injections, IV IL-2 administration, and palliative radiation. Ten dogs were treated with an average year of 8 years old, and all primary sites of disease were in the extremities. Four dogs were euthanized related to progressive osteosarcoma and two died of unrelated causes. Four dogs (40%) remained alive at 18 months [123,124]. This demonstrates the feasibility of NK cell immunotherapy as a viable treatment option for canine osteosarcoma patients and adds to the evidence that NK cell therapy is a new treatment option for pediatric/AYA osteosarcoma patients. 

## 5. Combination Therapy with Monoclonal Antibodies

GD2 is a ganglioside expressed throughout the central nervous system and is found on human neural stem cells, mesenchymal stem cells, peripheral nerve cells, melanocytes, and some solid tumors [129,130,131]. It is expressed on neuroblastoma and some other solid tumors such as melanoma, soft tissue sarcomas, osteosarcoma, desmoplastic small round cell tumor, and small cell lung cancer [131], though the expression is highest in neuroblastoma. Many monoclonal antibodies have shown success in treating adult solid tumors as monotherapy or combination therapy, pediatric solid tumor experience has lagged behind, with the notable exceptions of the anti-GD2 antibodies dinutuximab and naxitumab. These drugs kill tumor cells in an NK cell-dependent fashion by antibody-dependent cell-mediated cytotoxicity (ADCC) and NK cell independent-fashion by complement-mediated cytotoxicity (CMC). These effects can be augmented by coadministration with cytokines such as granulocyte-macrophage colony-stimulating factor (GM-CSF) and interleukin-2 (IL-2) [124]. Naxitumab (formerly humanized 3F8), another anti-GD2 monoclonal antibody, mediated peripheral blood mononuclear cell (PBMC) ADCC against neuroblastoma, which produced significant evidence of this killing mechanism over previous studies implicating oxidative lytic PMN cytotoxicity against target cells [117]. In contrast, dinutuximab (formerly ch14.18) was capable of lysing neuroblastoma cells with human granulocytes and NK cells [132]. To overcome human anti-mouse antibody (HAMA) responses, chimeric 3F8 (ch3F8) and humanized 3F8 (hu3F8) were produced, and compared to murine 3F8, both showed superior binding and more potent ADCC with similar biodistribution and cross-reactivity with other gangliosides [133]. Another human anti-GD2 antibody, hu14.18K322A, was designed with a point mutation inhibiting CMC and produced in rat myeloma line with less fucosylation to increase ADCC, thus decreasing pain and hypersensitivity reactions and increasing potency [123,127]. These modifications can potentially reduce side effects and prevent host reactions that would compromise the efficacy of the anti-GD2 treatment.

Both dinutuximab and naxitumab work best in the setting of minimal residual disease and show limited activity in the setting of bulky tumors. Combination therapy with NK cells may be a strategy to augment ADCC and is being explored in several clinical trials (Table 1). Lymphokine activated killers (which includes NK cells) from patients receiving IL-2 infusion, in combination with mouse or chimeric anti-GD2 antibody therapy, increased ADCC against neuroblastoma target cells, providing rationale for administering IL-2 in vivo to boost antitumor response [134]. Patients with high-risk neuroblastoma who were treated with ch14.18 in combination with IL-2 and GM-CSF with indication that the chemokine CXCL9 was somewhat prognostic at pretreatment, suggesting recruitment of immune effector cells like NK cells to the tumor could be beneficial [135]. 

To capitalize on the targeting capabilities of the anti-GD2 antibody and activating effects of IL-2, Gillies et al. developed an engineered fusion protein, hu14.18-IL-2, composed of the chimeric anti-GD2 antibody ch14.18 and IL-2 [136]. Comparing methods of delivery in a mouse neuroblastoma model, intratumoral hu14.18-IL-2 antitumor effect is driven by NK cells and T cells and increases tumor infiltrating T and NK activation, resulting in better inhibition of tumor growth and survival compared to intravenous immunocytokine [137]. Hu14.18-IL-2 is currently in clinical trials, in combination with haploidentical NK cells, for pediatric patients with relapsed neuroblastoma and osteosarcoma (NCT03209869).

Anti-GD2 antibody therapy may be affected by tumor expression of GD2 and the suppressive milieu of the tumor microenvironment. Transforming growth factor β (TGFβ) enhances tumor growth and survival and inhibits NK cell function. To oppose immunosuppressive effects, galunisertib, a small-molecule inhibitory of TGFβ receptor 1, was combined with dinutuximab and human activated NK cells in a neuroblastoma mouse model and showed significantly increased anti-tumor effect by reducing tumor growth [4]. In addition, efforts to overcome suppressive tumor microenvironments or immunologically “cold” tumors may enhance anti-GD2 antibody treatment. Applying radiation treatment and intratumoral immunocytokine to one single tumor of mice bearing multiple tumors causes suppressive regulatory T (T-reg) cell effects in untreated tumors curtailing in situ vaccination [138]. Thus, other approaches to combat these inhibitory effects are needed in the context of metastatic disease and immunosuppressive mechanisms. In a neuroblastoma mouse model, radiation and intratumoral anti-GD2 immunocytokine produced a potent antitumor response and resistance to re-challenge, suggesting efficacious in situ vaccination [139]. Further, addition of CpG and anti-CD40 to the regimen of radiation and intratumoral immunocytokine increased antitumor response, highlighting a role for macrophages, mobilizing effector T cells, and depleting regulatory T cells to augment treatment [139]. Approaches that mitigate tumor suppressive effects can prevent inhibition of antitumor response once effector cells reach the tumor microenvironment and promote tumor lysis and recognition of antigens to eliminate tumor at distant sites.

Studies have also investigated the combination of monoclonal antibodies with adoptive cell transfer. Infusion of activated cells would potentially facilitate trafficking of effectors to tumor sites and overcome the suppressive effects of existing tumor. Activated human NK cells combined with dinutuximab show increased cytotoxicity against neuroblastoma in vitro and after tumor resection in a neuroblastoma mouse model [140]. To boost the cytotoxic profile of effector cells, ex vivo IL-2 and IL-7 expanded natural killer-T lymphocytes were combined with anti-GD2 antibody to target human neuroblastoma cells and had significantly better tumor cell killing than monotreatment conditions in vitro [141]. As indicated by studies demonstrating benefit with the missing KIR ligand, anti-GD2 therapy could induce ADCC more effectively with KIR mismatch in which NK cells are not inhibited by the presence of self-HLA on target tumor, which could be accomplished with infusion of allogeneic NK cells that are KIR mismatched from the recipient. In a phase I study, murine 3F8 in combination with haploidentical NK cells was administered to patients with high-risk neuroblastoma, and 29% of patients had a complete or partial remission [142]. No GVHD was seen and circulating NK cells were undetectable beyond 2 weeks after infusion, despite severe lymphodepletion [142]. In a pilot trial, 13 patients with recurrent/refractory neuroblastoma were treated with hu14.18K322A, GM-CSF, IL-2, and chemotherapy combined with an infusion of haploidentical NK cells resulting in a 61.5% response rate and 38.5% had stable disease [143]. A study combining data from two prospective clinical trials evaluated pediatric patients with refractory or relapsed neuroblastoma treated with allogeneic HSCT and included a few patients that had received anti-GD2 therapy but did not compare outcomes of these patients to those that did not receive anti-GD2 therapy [144]. Further studies are needed to optimize generation of NK cells and combinations of adoptive cell therapies or allogeneic transplant with anti-GD2 antibodies. 

## 6. CAR NK Cells

Chimeric antigen receptors (CARs) are synthetic immune cell receptors that are engineered to grant antibody specificity to a particular cell surface antigen tethered with the signaling machinery that programs a cell to kill the target cell bearing the antigen [145]. CD19 targeted CAR T cell therapies have achieved up to 90% remission in patients with relapsed or refractory B-cell lymphoblastic leukemia [146]. NK cells lack the appropriate machinery to recognize tumor antigens without binding Fc-gamma receptors through CD16 to IgG-coated targets, however introducing a CAR to NK cells can overcome this limitation. Early clinical studies in adults with chronic lymphocytic leukemia have shown that CAR NK cells are safe and effective, though proof of efficacy outside of targeting CD19 has been inconsistent between in vivo studies and clinical trials [147]. Still, CAR design for NK cells has progressed rapidly and newly designed CAR-NK cells expressing IL-15 have shown promising results.

A CAR with NKG2D fused to the T cell receptor ζ-chain expressed in NK cells demonstrated cytotoxicity against myeloid-derived suppressor cells and improved antitumor activity against neuroblastoma [148]. Primary CAR NK cells have been doubly transduced with a rapamycin inducible chimeric gene encoding myeloid differentiation primary response gene 88 (MyD88), a critical adaptor protein in the signal transduction of the TLR signaling pathway, and the costimulatory protein CD40 [28]. The combination, name iMC, was combined with an inducible caspase-9 safety switch, and a CD123-directed CAR. The 4 gene combination was successfully expressed in K562 expanded primary NK cells with lentiviral transduction (69.02% efficiency) and in vitro and in vivo studies showed that the CAR NK cells significantly controlled tumor growth when the iMC switch was activated. The study was repeated with a B cell maturation antigen (BCMA)-directed CAR in which expression of the CAR was greater than 40% and the BCMA CAR significantly controlled tumor growth, similar to the CD123 CAR proposing an innovative “dual switch” approach to addressing current limitations of CAR NK cell efficacy and persistence. Liu and colleagues compared the efficacy between NK cells that were collected from cord blood (CB) or chronic lymphocytic leukemia (CLL) patients and retrovirally transduced to express an anti-CD19 CAR, alongside IL-15 and a suicide gene, inducible caspase-9. They found that the CB derived CD19-CAR NK cells performed better than non-transduced (NT) CB derived NK cells and autologous CD19-CAR NK cells derived from the CLL patients themselves. Inhibiting NKG2A/HLA-E interaction with a NKG2A blocking antibody significantly improved the ability of patient-derived NT NK cells, patient derived CD19-CAR NK cells, and CB CD19-CAR NK cells to kill primary CLL cells, however CB CD19-CAR NK cells consistently outperformed all other NK cell treatments with or without NKG2A blockade [149]. These data reveal that the incorporation of IL-15 CAR constructs, allogeneic sources of NK cells, and checkpoint blockade may be viable approaches for improving the cytolytic function of CAR NK cells. The CB NK cells that were engineered to express anti-CD19 CAR, IL-15, and an inducible caspase 9 safety switch were used in a phase 1 and 2 trial in 11 patients with relapsed or refractory CD19-positive cancers. Of the 11 patients treated with 1 dose of CAR NK cells, 8 patients (73%) responded including 7 patients who had a complete response and 1 patient who had a complete remission of high-grade lymphoma [150]. None of the patients experienced cytokine release syndrome, neurotoxicity or GVHD and none of the cases required activation of the caspase 9 safety switch thus displaying the safety of allogeneic CAR NK cells.

Other phase I and II CAR-NK trials have been completed; these have been primarily targeted to hematologic malignancies [138]. Few studies have been completed in solid tumors (NCT02839954, NCT03656705, and NCT03383978), though none have yet been studied in pediatrics. A recent clinical trial (NCT03294954) utilized autologous CAR natural killer T (NKT) cells with co-expression of GD2 and IL15 for patients with relapsed or refractory neuroblastoma. Interim analysis of the first three patients treated with 3 × 10^6^ ex vivo expanded CAR NK cells showed no dose limiting toxicities, suggesting NKT cells can be successfully expanded and used to treat pediatric cancers [151]. The technology driving CAR NK cells continues to advance resulting in added sources for NK cells and dynamic CAR engineering. Sources for NK cells have expanded beyond the patient and their relatives to allogeneic NK cells derived from the NK92 cell line, peripheral blood mononuclear cells (PBMCs) of healthy donors or umbilical cord blood (UCB), and iPSCs for an “off-the-shelf” cell therapy product [27]. Furthermore iPSC-CAR NK cells have shown great promise in preclinical studies, proving to be just as effective as CAR NK cells derived from peripheral blood [152]. iPSC-CAR NK cells performed and NK-92s but possess the advantage of not requiring irradiation [153]. Thus, new developments in CAR NK cell design and manufacturing will result in an adoptive NK cell therapy with improved engraftment, expansion, persistence, and performance in the clinical setting.

## 7. Clinical Trials Involving NK Cell Enriched Allogeneic HSCT for Pediatric Solid Tumors

Given pediatric solid tumors’ poor response to traditional therapies, many studies have examined allogeneic HSCT as a salvage option. While autologous HSCT is the standard of care for diseases such as neuroblastoma, allogeneic HSCT has the added potential benefit of the graft vs. tumor (GVT) effect. While transplants for leukemia are often HLA-matched to reduce the risk of GVHD, haploidentical transplants offer a unique opportunity for mismatching donor NK cells (specifically inhibitory KIR molecules) against HLA molecules present on the tumor, enhancing the GVT effect [154]. As GVHD is T-cell mediated, there is increased interest in administering other effector cells like NK cells to improve the GVT effect while minimizing GVHD [4,155,156]. In adult oncology, early use of allogeneic HSCT for renal cell carcinoma demonstrated GVT effect, though it was complicated by high rates of morbidity and mortality following transplant, along with a lack of durable response [157,158]. Another significant complication has been GVHD, which does correlate with GVT in leukemias but has not shown any benefit to patients with solid tumors. GVHD is a limiting factor in using allogeneic transplant for treatment of solid tumors [155], NK cells present a viable treatment option that minimizes GVHD while maintaining GVT effect [4,139].

Llosa et al. conducted a study examining haploidentical HSCT for pediatric and young adult patients with solid tumors in relapse. They included patients with DSRCT, neuroblastoma, rhabdomyosarcoma, Ewing’s sarcoma, PNET, and pineoblastoma. All patients had received previous therapies and were in anywhere from a complete response (CR) to progressive disease (PD) at study entry. Overall survival (OS) was 88, 56, and 21% at 6, 12, and 24 months, respectively. While the trial showed feasibility and safety of transplant in this population, median survival was 14 months [159].

While the risk of GVHD is increased with haploidentical HSCT compared to HLA-matched HSCT, grafts infused with higher percentages of NK cells have been reported to have lower rates of GVHD [134]. Lang et al. examined usage of haploidentical HSCT with T and B cell depleted grafts, with the majority of transplanted cells being NK cells. In that study, six patients with relapsed neuroblastoma or sarcomas (rhabdomyosarcoma or Ewing’s sarcoma) received a melphalan based, reduced intensity conditioning regimen. Despite a heavy tumor burden and extensive pretreatment (including autologous stem cell transplants), patients tolerated transplant. However, median survival was still only 6 months in this cohort. This demonstrated the feasibility of NK cell-enriched haploidentical transplants, but also showed the need for further immunomodulation following transplant.

A current study at University of Wisconsin uses alpha/beta T cell and CD19+ B cell depleted haploidentical HSCT to reduce the risk of GVHD (NCT02508038) to treat children with relapsed/refractory solid tumors. This study uses zoledronate as immunomodulation for five doses following transplant. In addition to the known effects of activating gamma-delta T cells [160], zoledronate activates NK cells in a dendritic cell dependent manner and leads to increased IFN-γ production [161]. This suggests the zoledronate may play a role in activating NK cells post-transplant. Pediatric patients receiving haploidentical TCRαβ+ and CD19+ depleted HSCT, retaining TCRγδ and NK cells in the graft, were treated with zoledronate, which was well tolerated with rapid engraftment and low GVHD toxicity [162].

## 8. Adoptive NK Cell Trials for Pediatric Solid Tumors

Experience with HSCT and monoclonal antibodies has led to increased testing of NK cells alone or in combination with other immunotherapy for pediatric solid tumors. NK cells have been shown to be largely reduced or have impaired activity in many newly diagnosed oncology patients [117,163]. For example, in osteosarcoma, NK cells showed intact functionality and IFN-y production, but in low numbers [164]. For patients treated with chemotherapy, early recovery of lymphocyte counts conferred improved survival [165]. These findings support the theory that NK cells are critical to survival in osteosarcoma patients and suggests that NK cell infusions are viable treatment options to replace impaired endogenous NK cells.

A current phase 2 trial at Children’s Hospital of Wisconsin (STIR trial, NCT02100891), recently completed recruiting patients with high-risk solid tumors including Ewing’s sarcoma, rhabdomyosarcoma, osteosarcoma, neuroblastoma, and CNS tumors. An additional NK trial is currently active at MD Anderson (NCT03420963) for pediatric solid tumors. This trial uses non-myeloablative conditioning with cyclophosphamide and etoposide, prior to receiving cord-blood derived ex-vivo expanded allogeneic NK cells. Differing from other studies, this uses HLA matched NK cells derived from cord blood. This phase 1 trial may reduce the GVHD seen in prior studies from haploidentical donors. 

## 9. Combination Therapy with NK Cells and Immunotherapies

Considering NK cell involvement with ADCC, their phenotype may influence the efficacy of anti-GD2 therapy. Stochastic expression of KIRs determine not only NK cell function via activating and inhibitory signals, but also influence clinical outcome. Genotyping of patients with neuroblastoma treated with dinutuximab, GM-CSF, IL-2, and isotretinoin revealed that certain inhibitory KIR/KIR ligand genotypes had better event-free survival (EFS) and OS, but those with KIR ligand missing for their inhibitory KIRs did not show benefit with immunotherapy over isotretinoin alone [166]. In neuroblastoma patients treated with 3F8 mouse anti-GD2, missing KIR ligand, lacking one or more HLA class I ligand for their inhibitory KIR, was associated with increased OS and progression-free survival (PFS), and in vitro unlicensed NK cells are spared from inhibition by cytokine-induced expression of self-HLA on tumor cells [70]. Thus, missing KIR ligand may be a positive prognostic factor for patients treated with anti-GD2 antibody.

Combination therapy with NK cells and anti-GD2 antibodies has been used in several clinical trials to date for neuroblastoma, with new trials opening for osteosarcoma in the near future. A pilot study of parentally derived NK cells (cycles 2, 4, and 6) with antiGD2 antibody hu14.18K322A, plus chemotherapy cyclophosphamide/topotecan (courses 1 and 2), irinotecan/temozolomide (courses 3 and 4), and ifosfamide/carboplatin/etoposide (courses 5 and 6) was used to treat 13 patients with relapsed/refractory neuroblastoma. All the patients were heavily pre-treated. Response rate was 61.5% (four complete responses, one very good partial response, and three partial responses) and five had a stable disease, with 77% OS at 1 year [167]. A similar phase 1 trial combining a different anti-GD2 monoclonal antibody, 3F8, combined with haploidentical NK cells follow lymphodepleting chemotherapy enrolled 35 patients. The primary goal was to determine maximum tolerated NK dose. Doses ranged from <1 × 10^6^ to 50 × 10^6^. Results did not show a high a response rate, possibly due to the low starting dose. However, improved EFS were seen in patients receiving >10 × 10^6^ NK cells [168]. Several other trials are in process at this time (Table 2). These findings suggest that NK cells can be safely combined with anti-GD2 antibodies, and may show anti-neuroblastoma effects, especially at higher NK cells doses. 

## 10. Conclusions

NK cells show great potential alone or in combination for treatment of pediatric solid tumors. As a liaison between the innate and adaptive immune system, NK cells rely on highly regulated antigen-independent mechanisms to distinguish between healthy cells and malignant cells. Their varied but potent killing mechanisms can be leveraged to target cancers that exploit antigen shedding, downregulation of MHC-I, or T cell exhaustion to evade adaptive immune surveillance. The capacity for NK cells to be the next breakthrough cell therapy in cancer immunotherapy is certainly present as NK cells are capable of mobilizing the adaptive immune system in addition to mediating direct tumor lysis. NK cells exhibit this potency without driving GVHD, provide a shorter acting effector cell compared to T cells, and have shown promising results in terms of their safety profile and preclinical efficacy assays. Incorporating NK cells in new approaches to treating pediatric solid tumors presents an appealing opportunity to improve clinical outcomes. In order to bring adoptive NK cell therapy to pediatric solid tumors in the clinical setting, NK cells must show a consistent and significant benefit in both in preclinical studies and in clinical trials. While early clinical trials have shown that NK adoptive cell transfer can be done safely in pediatric patients with relapsed solid tumors, improved outcomes have not yet been consistently seen in this population. However, advances in our understanding of NK cell biology, checkpoint inhibition, CAR technology, and expansion of autologous and allogeneic NK cells continue to push adoptive NK cell-based therapies forward as a highly activated cellular therapeutic for pediatric cancer.

## Figures and Tables

**Table 1:** Current antibody therapies that may enhance NK-mediated antitumor mechanisms.

Target	Indications	Therapy	Drug Type	Status
GD2 [108]	Melanoma, neuroblastoma, osteosarcoma	3F8	Mouse IgG3	Phase I, II
		ch14.18 (Dinutuximab)	Mouse human chimeric IgG1	FDA approved (neuroblastoma)
		hu14.18-IL2	Humanized 14.18 fused with IL-2	Phase I, II
		hu14.18K322A	Point mutation in hu14.18	Phase I, II
		hu3F8 (Naxitumab)	Humanized 3F8	FDA approved (neuroblastoma)
NKG2A [91]	NSCLC, head and neck squamous cell carcinoma, colorectal cancer, gynecologic cancers, other advanced solid malignancies	IPH2201 (Monalizumab)	Humanized IgG4	Phase I, II
KIR2D [109]	Breast, kidney, and ovarian carcinomas	IPH2102 (alirocumab)	Human IgG4	Phase I
TIGIT [92,110,111]	Advanced solid tumors	OMP-313M32 (Etigilimab)	Human IgG1	Phase I
	NSCLC, SCLC, esophageal cancer, advanced solid tumors	MTIG7192A/RG-6058 (Tiragolumab)	Human IgG1	FDA approved (NSCLC)
	Melanoma	MK-7684 (Vibostolimab)	Humanized IgG1	Phase I, II
	Multiple myeloma	BMS-986207	Human IgG1	Phase I
	Advanced solid tumors	ASP-8374	Human IgG4	Phase I
	Metastatic solid tumors	BGB-A1217	Humanized IgG1	Phase I
	NSCLC, advanced solid tumors	AB-154	Humanized IgG1	Phase I
TIM-3 [98,110]	Liver cancer, advanced solid tumors	TSR-022	Human IgG4	Phase I
	Advanced malignancies, AML, MDS	MBG453	Humanized IgG4	Phase I, II
	Solid tumors and lymphomas	SYM023	Human IgG1	Phase I
	Solid Tumors	INCAGN2390	Human IgG1	Phase I
	Advanced solid tumors	LY3321367	Human IgG1	Phase I
	Advanced malignancies	BMS-986258	Human IgG1	Phase I
	Solid Tumors	BGBA425	Humanized IgG1	Phase I
	Advanced solid tumors	SHR-1702 (Camrelizumab)	Humanized IgG4	Phase I
	Melanoma, NSCLC, solid tumors	RO7121661	Bi-specific antibody targeting PD-1 and TIM-3	Phase I
DNAM-1 [92]	Advanced solid tumors	LY3435151	DNAM-1 agonist	Phase I
LAG-3 [112,113,114,115]	Breast carcinoma, melanoma, RCC, solid tumors	IMP321 (Eftilagimod alpha)	LAG-3 IgG1 Fc fusion protein	Phase I, II
	Advanced solid tumors, melanoma, colon cancer, hematologic malignancies, glioblastoma, gliosarcoma, advanced gastric cancer, NSCLC, RCC	BMS-986016 (Relatlimab)	Human IgG4	Phase II, III
	Advanced solid tumors, advanced hematologic malignancies	LAG525	Humanized IgG4	Phase I, II
	Advanced solid tumors, hematologic malignancies	MK-4280	Humanized IgG4	Phase I, II
	Malignancies	REGN3767	Human IgG4	Phase I
	Advanced solid tumors	TSR-033	Humanized IgG4	Phase I, II
	Advanced solid tumors, cholangiocarcinoma, liver cancer, gastric cancer, breast cancer, esophageal cancer, hematologic malignancies	MGD013	Bi-specific DART^®^ IgG4k antibody targeting PD-1 and Lag-3	Phase I
	Advanced malignancies	FS118	Bi-specific antibody targeting PD-L1 and LAG-3	Phase I
	Advanced solid tumors, lymphomas	Sym022	Human Fc-inert	Phase I
	Solid tumors	XmAb22841	Bi-specific Fc-inert antibody	Phase I
B7-H3 [110]	Solid tumors, pediatric B7-H3 expressing solid tumors, prostate cancer	MGA271 (Enblituzumab)	Humanized IgG1	Phase I, II
	Advanced solid tumors	MGD009	Bi-specific DART^®^ IgG1 antibody targeting B7-H3 and CD3	Phase I
	DSRCT, advanced CNS or leptomeningeal cancer, neuroblastoma, gliomas	131I-8H9 (Omburtamab)	Murine IgG1	Phase I

AML—acute myeloid leukemia, CNS—central nervous system, DSRCT—desmoplastic small round cell tumors, MDS—myelodysplastic syndrome, NSCLC—non-small cell lung cancer, RCC—renal cell carcinoma, SCLC—small cell lung cancer.

**Table 2:** Current pediatric clinical trials including adoptive NK cell therapy.

Clinical Trial Number	Phase	Title	Sponsor Institution	Status	Condition Treated	Trial Goals	Treatment Notes
NCT00582816	1	Haploidentical Transplant with NK Cell Infusion for Pediatric Acute Leukemia and Solid Tumors	University of Wisconsin, Madison	Terminated (toxicity)	Relapsed/refractory leukemia or solid tumors	Primary outcomes: GVHD, engraftment failure, number of days until engraftment criteria were met, mortality rate	Methylprednisolone, Equine ATG, Cyclosporine, Fludarabine, Melphalan, Thiotepa and Rituximab
NCT01875601	1	NK White Blood Cells and Interleukin in Children and Young Adults with Advanced Solid Tumors	National Cancer Institute (NCI)	Completed	Relapsed/refractory solid tumors	Primary objectives are: (1) to assess the feasibility of harvesting and expanding activated NK cells to meet escalating dose goals in Cohort A, (2) to assess the toxicity of infusing escalating doses of activated NK cells following lymphodepleting chemotherapy without rhIL15 (cohort A), and (3) to assess the toxicity of infusing NK activated cells with escalating doses of rhIL15 (cohort B) in pediatric patients with refractory malignant solid tumors.	All patients receive pre-NK lymphodepleting chemotherapy with cyclophosphamide. Cohort A receives escalating doses of NK cells; Cohort B receives escalating doses of NK cells and rhIL15
NCT00640796	1	Pilot Study of Expanded, Donor Natural Killer Cell Infusions for Refractory Non-B Lineage Hematologic Malignancies and Solid Tumors	St. Jude Children’s Research Hospital	Completed	Relapsed/refractory hematologic malignancies, Ewing sarcoma family of tumors (ESFT) and rhabdomyosarcoma (RMS)	To determine the maximum tolerated dose of expanded NK cells in research participants with relapsed or refractory hematologic malignancies and sarcomas.	Haploidentical NK cells + Cyclophosphamide, Fludarabine, Interleukin-2, Mesna
NCT02130869	1	A Pilot Study of Immunotherapy Including Haploidentical NK Cell Infusion Following CD133+ Positively-Selected Autologous Hematopoietic Stem Cells in Children with High-Risk Solid Tumors or Lymphomas	St. Jude Children’s Research Hospital	Completed	Relapsed/refractory neuroblastoma, lymphoma, high risk solid tumor	To evaluate day +35 absolute neutrophil count (and) engraftment in autologous stem cell transplantation for high-risk pediatric malignancies after stem cell selection and immunotherapy.	All participants first receive standard of care high-dose chemotherapy specific to their tumor type. Group C participants receive melphalan, etoposide (or etoposide phosphate), carboplatin, CD133+ selected autologous stem cell infusion, IL-2, haploidentical natural killer cell infusion, G-CSF, and GM-CSF.
NCT02100891	2	Phase 2 STIR Trial: Haploidentical Transplant and Donor Natural Killer Cells for Solid Tumors (STIR)	Medical College of Wisconsin	Active, not recruiting	Relapsed/refractory neuroblastoma, Ewing sarcoma, rhabdomyosarcoma, osteosarcoma, CNS tumors	Disease-control rate	Patients will receive a reduced-intensity conditioning regimen for 6 days that consists of Fludarabine 150 mg/m^2^, Cyclophosphamide 29 mg/kg, and 3 Gy total body irradiation (TBI), followed by HLA-haploidentical marrow from a family member on Day 0. On Days +3 and +4, Cyclophosphamide 50 mg/kg will be infused for selective in vivo T cell depletion. Additional post-grafting immune suppression will consist of mycophenolate mofetil and either tacrolimus or sirolimus. PBMCs will be collected from donors on Day +6, from which NK cells will be selected and infused into patients on Day +7.
NCT02508038	1	Alpha/Beta CD19+ Depleted Haploidentical Transplantation + Zometa for Pediatric Hematologic Malignancies and Solid Tumors	University of Wisconsin, Madison	Recruiting	Relapsed/refractory leukemia, lymphoma, neuroblastoma, Ewing sarcoma, rhabdomyosarcoma, osteosarcoma, PNET	Incidence of acute GVHD. Incidence of graft failure	Patients with high-risk leukemia will receive myeloablative conditioning with anti-thymocyte globulin intravenously (IV) over 4–6 h on days −12 through −9, Fludarabine IV over 30 min on days −8 through −5, Thiotepa IV every 12 h on day −4 and total body irradiation (TBI) on days −3 through −1. All other patients receive reduced intensity conditioning consisting of anti-thymocyte globulin intravenously (IV) over 4–6 h on days −12 through −9, fludarabine IV over 30 min on days −8 through −5, thiotepa IV over 4 h every 12 h on day −4, and melphalan IV on days −3 and −2. Patients undergo TCR-alpha/beta+ and CD19+ depleted KIR/KIR ligand-mismatched haploidentical donor PBMC transplantation on day 0.
NCT01576692	1	Combination Chemotherapy, Monoclonal Antibody, and Natural Killer Cells in Treating Young Patients with Recurrent or Refractory Neuroblastoma	St. Jude Children’s Research Hospital	Completed	Relapsed/refractory neuroblastoma	To observe and describe the toxicities associated with humanized anti-GD2 antibody (hu14.18K322A) with and without allogeneic NK cells when given with repeated cycles of chemotherapy to children with refractory/relapsed neuroblastoma.	A maximum of 6 courses of therapy may be given on the following schedule: Courses 1, 3, and 5: Humanized anti-GD2 antibody + chemotherapy. Courses 2, 4, and 6: Humanized anti-GD2 antibody + chemotherapy, with or without natural killer (NK) cells (depending on availability of appropriate NK donor). NK Cell dosage: minimum of 0.1 × 10^6^ cells/kg; maximum of 400 × 10^6^ CD45+ cells/kg, given once
NCT03209869	1	Treatment of Relapsed or Refractory Neuroblastoma and Osteosarcoma with Expanded Haploidentical NK Cells and Hu14.18-IL2	University of Wisconsin, Madison	Suspended (COVID)	Relapsed/refractory neuroblastoma and osteosarcoma	Safety: Incidence of treatment-emergent adverse events of treatment with AENK cells and hu14.18-IL2	All subjects will receive Ex vivo Expanded and Activated Haploidentical Donor NK Cells + hu14.18-IL2
NCT00877110	1	Anti-GD2 3F8 Antibody and Allogeneic Natural Killer Cells for High-Risk Neuroblastoma	Memorial Sloan Kettering Cancer Center	Completed	Relapsed/refractory neuroblastoma	Assess the feasibility and safety of administering allogeneic haploidentical NK infusions with 3F8 in patients with high-risk NB	Patients will receive combination chemotherapy with intravenous cyclophosphamide for two days, IV vincristine for one day, and IV topotecan 2.4 mg/m^2^/day for 3 days.
NCT02650648	1	Humanized Anti-GD2 Antibody Hu3F8 and Allogeneic Natural Killer Cells for High-Risk Neuroblastoma	Memorial Sloan Kettering Cancer Center	Active, not recruiting	Relapsed/refractory neuroblastoma	The number patient responses observed at each dose level	Following chemotherapy, three dose levels of NK cells, starting at dose level 1, will be evaluated in this treatment protocol. Cyclophosphamide will be given for two days (days −6 and −5). On Days −1, +1, +5, +7, and +9 hu3F8 is administered. On day 0, daily from +2 through +4, day +6, and day +8, rIL-2 is administered subcutaneously.
NCT02573896	1	Immunotherapy of Relapsed Refractory Neuroblastoma with Expanded NK Cells	New Approaches to Neuroblastoma Therapy Consortium	Recruiting	Relapsed/refractory neuroblastoma	Feasibility of expanding NK cells from neuroblastoma patients and cryopreserving, shipping, and infusing multiple doses of NK cells	NK cells on Day 5 and 17.5 mg/m^2^/dose of Ch14.18 on Day 1–4. Patients will also receive 25 mg/m^2^/dose of Lenalidomide during Day −6 through 14 of treatment.
NCT00698009	1	Haploidentical Natural Killer (NK) Cells in Patients with Relapsed or Refractory Neuroblastoma	M.D. Anderson Cancer Center	Terminated (slow accrual)	Relapsed/refractory neuroblastoma	Participant Disease Response	Fludarabine 25 mg/m^2^ intravenous daily starting 6 days before the NK cell infusion (considered Day −6) and once a day through Day −2. Cyclophosphamide 60 mg/kg IV days −5 and −4. Natural Killer Cell Infusion on Day 0.
NCT03294954	1	GD2 Specific CAR and Interleukin-15 Expressing Autologous NKT Cells to Treat Children with Neuroblastoma (GINAKIT2)	Texas Children’s Hospital	Recruiting	Relapsed/refractory neuroblastoma	Maximum tolerated dose of autologous NKTs expressing a 2nd generation GD2-specific chimeric antigen receptor administered to patients with relapsed or refractory neuroblastoma	GINAKIT cells + Cytoxan + fludara
NCT01287104	1	A Phase I Study of NK Cell Infusion Following Allogeneic Peripheral Blood Stem Cell Transplantation from Related or Matched Unrelated Donors in Pediatric Patients with Solid Tumors and Leukemias	National Cancer Institute (NCI)	Completed	Relapsed/refractory leukemia or solid tumors	To assess the feasibility and toxicity of infusing escalating doses of donor-derived activated NK cell donor lymphocyte infusions (NK-DLI) on Days 7 plus or minus 2 days and 49 plus or minus 7 days following human leukocyte antigen (HLA)-matched T cell depleted (TCD) peripheral blood stem cell transplant (PBSCT)	A phase 1 cell dose escalation of donor derived NK-DLI will be performed using 3 dose levels infused on days 21 more or less 3 post-PBSCT and a second infusion on day 49 more or less 7 post-PBSCT.
NCT03420963	1	Donor Natural Killer Cells, Cyclophosphamide, and Etoposide in Treating Children and Young Adults with Relapsed or Refractory Solid Tumors	MD Anderson	Recruiting	Relapsed/refractory solid tumors	Determine the safety, maximum tolerated dose and/or recommended phase II dose of cord blood-derived expanded allogeneic natural killer cells following chemotherapy	Patients receive cyclophosphamide IV QD over 30 min and etoposide IV QD over 60 min on days 1–5 in the absence of unacceptable toxicity. Patients then receive cord blood derived allogeneic NK cells IV on day 8.
NCT04211675	1/2	A Phase I-II Study of Ex-Vivo Expanded Autologous NK Cells Infusions in Combination with Irinotecan, Temozolomide, and Dinutuximab in Patients with Relapsed or Refractory Neuroblastoma: The STING Trial	Nationwide Children’s Hospital	Not yet recruiting	Relapsed/refractory neuroblastoma	NK cells safety and tolerability: Number of participants with treatment-related adverse events and toxicities. Response to NK Cell treatment as determine by CT/MRI imaging, MIBG imaging, and bone marrow aspiration.	6 cycles of 21 days each consisting of irinotecan, temozolomide, Dinutuximab, and Sargramostim (Cycle 1), or irinotecan, temozolomide, Dinutuximab, Sargramostim, and natural killer (NK) cells (Cycles 2–6).
NCT02409576	1/2	Pilot Study of Expanded, Activated Haploidentical Natural Killer Cell Infusions for Sarcomas (NKEXPSARC)	National University Hospital, Singapore	Recruiting	Relapsed/refractory Ewing sarcoma, rhabdomyosarcoma	Disease response after expanded activated NK cell infusion	Day −7 Cyclophosphamide at 60 mg/kg. Day −6 Fludarabine at 25 mg/m^2^ daily for 5 days. Each patient will receive radiation within 48 h of NK cell infusion to make the tumor cells more sensitive to NK cell killing Radiation 2 Gy. Each patient will receive IL-2 to support NK cell activation and expansion in vivofor a total of 6 doses. Expanded activated haploidentical NK cells will be infused on day 0.
NCT04214730	N/A	Study of Natural Killer Cell Combined with Chemotherapy for Advanced Solid Tumor	Yantai Yuhuangding Hospital	Recruiting	Relapsed/refractory solid tumors	Disease Control Rates	Patients in group A will receive 4 cycles of NK treatments within 8 months. Patients in group B will have no immunotherapy. Chemotherapy is available in both groups.
